# A Short History by the Viscountess Cowdray
*Given at the Liverpool meeting of the College of Nursing, February 22, 1918.


**Published:** 1918-03-16

**Authors:** 


### THE BRITISH WOMEN'S HOSPITAL COMMITTEE.

### A Short History by the Viscountess Cowdray.*

I have always been a patroness of this Society and have subscribed to their funds. They assured me that if I would come and take charge of the money, as they felt that would be a sufficient guarantee in the country that it was well looked after, they could do a great deal of work. Without my help they said it was utterly impossible, beause they worked three months in London and three months in the country, and so on, so that they must have somebody who was always in London. I have been in London ever since the war began, without any break, to watch over things and to look after the money, which is my responsibility.

Let me explain as simply as possible how we do our work. The "Star and Garter" has been a great joy to us. The public were anxious to have a memorial to our brave men, and when we were asked, why, of course, we imagined we were going to have that memorial very quickly, that the war would end quickly, and that we would be able to get it, and to get it almost immediately. That, of course, has not been possible of accomplishment; but temporary buildings have been employed and the work for the men is being done admirably. When that was finished, we felt how delightful it would be if we could help in making a permanent memorial to the women of the country, as we had been so successful in securing one for the men. I hope it will be believed that that was at least a good wish of the committee generally.

We started on the work with great pleasure and determination. We hesitated for three months, because we had opposition brought to bear, and we felt it was only right that we should all be quite convinced, entirely convinced, before we went on with the work. I who have been interested in nursing for the last thirty years knew perhaps more about it than the rest of the committee; but they all became convinced and keen, and so we are at work. We are thankful to feel that there are now 8,000 members on the College Register, and our one regret is that we have not the 80,000 nurses in the country with us entirely. But I do not doubt that in the first ten years of the College they may at least be expected to get 40,000, which will be one-half, and I certainly have no doubt that in the second ten years the whole of the nursing profession will be members of the College, because the nursing profession will by then have realised the benefit that the College is to them as a profession and as individuals.

We are sometimes told, "Why interfere with the nursing profession?" I should like you to understand that we are not interfering, we are only anxious to help. It was not "interfering" when the country helped the soldiers, or when they endowed their Colleges, and we consider it is only help, that the nursing profession is fully deserving, and that we are attempting by means of the Nation's Fund for Nurses to supply that help. The nursing profession is the most unselfish body in the whole of the world. I do not hesitate to say that for one moment. I have had experience of nurses in the Queen's nurses. I also founded a small hospital in Mexico City, where I had the whole of the work to do, so that I am not quite ignorant of nurses and nurses' requirements. I know that the unselfishness of nurses, the long hours of their work, and the difficulty of finding time for study during their training are a serious drawback to the profession. I remember when I went from Mexico City to Washington to inquire from the head of the Red Cross there the best lines on which to work my nursing home and the visiting nurses and districts, being impressed with the efficiency of the nursing in Washington, the efficiency of the operating theatres, and all the magnificent arrangements. They were all of great interest to me. I spent several weeks going to the different private hospitals belonging to my friends, to the public hospitals, and the places where all the instruments are made. It was a revelation. We have nothing in this country like it. I do not say that our nurses are not equal; they are superior, taking them as a whole, to the American nurses; but the Americans make a speciality of things. I want the College to be able to give their nurses more opportunity, more educational opportunity, of seeing what is being done in other countries. The very arrangements and the very way that everything is done should be the standard that it is done everywhere in the best possible manner. The education of nurses is sometimes spoken of. In many hospitals it is very difficult for nurses to get time to study. I have had a good deal to do with the South London Hospital, which is run entirely by women, doctors and nurses. The Royal Free Hospital is another hospital I have always been very much interested in, as well as the London Hospital, and a great many other hospitals in London. I think it should be compulsory that nurses should have time given to them for study. There is a great deal of truth in the saying that "nurses are born, not made," and I believe that if there were scholarships in the schools whereby girls could be brought out and helped in their training as nurses, it would be a benefit to the country. In the future reconstruction after the war there will no doubt be a re-establishment in many branches, and we want at least to be sure that we have learned something from this terrible war. We must learn to be more efficient in everything—more efficient in education, more painstaking, more economical, and more determined, to have the best always, because the best is the cheapest. If that is done, all must see that we feel ourselves justified in struggling to put the nursing profession on to a firmer foundation. And because it is for the moment a ship rather in troubled waters I do not feel that I should be the right kind of captain of finance of the Nation's Fund for Nurses if I were to leave the ship because it is struggling a little. Indeed, if we have right on our side we need not fear anything. Let us, however, be quite sure that we have right, and then all must in the end go well.

* Given at the Liverpool meeting of the College of Nursing, February 22, 1918.

